# Creating an Age-Friendly Model System Through a Community and University Partnership

**DOI:** 10.1093/geroni/igab046.378

**Published:** 2021-12-17

**Authors:** Andrew Revell, Jennifer Viveiros

**Affiliations:** 1 University of Massachusetts Dartmouth, University of Massachusetts Dartmouth, Massachusetts, United States; 2 University of Massachusetts Dartmouth, North Dartmouth, Massachusetts, United States

## Abstract

The University of Massachusetts 5-campus system was the first university system to receive the Age-Friendly University designation in the AFU Global Network (Business West, 2019). Simultaneously, the town of Dartmouth and city of New Bedford became Age-Friendly Communities. This allowed for dynamic collaboration between our university and communities. This presentation highlights several examples. The Ora M. DeJesus Gerontology Center faculty and student researchers developed the original age-friendly survey items for New Bedford’s initial community assessment; and the College of Nursing and Health Sciences faculty and student researchers compiled data for Dartmouth’s survey. Community service during the pandemic has flourished. The Community Companions program, which matches students with community members in social need, went virtual. Nursing students and faculty have been on the frontline in the vaccination efforts in the town of Dartmouth. These partnerships will be presented as examples of potential opportunities for other age-friendly communities. Community-university partnerships are encouraged.

